# Resident autonomy in orthopedic trauma: independent femoral IMN by senior residents yields comparable outcomes to fellowship-trained surgeons

**DOI:** 10.1007/s00402-026-06460-8

**Published:** 2026-08-24

**Authors:** Snir Balziano, Gilad Nesher, Nitzan Ben Nun, Tomer Segev, Alon Freidlander, Dan Prat

**Affiliations:** 1 Sheba Medical Center, Ramat Gan, Israel; 2 Tel Aviv University, Tel Aviv, Israel

**Keywords:** Resident autonomy, Orthopedic trauma, Femoral intramedullary nailing, Senior residents, Surgical outcomes

## Abstract

**Introduction:**

Intramedullary nailing (IMN) of femoral shaft fractures is a fundamental cornerstone procedure in orthopedic trauma and an important benchmark of resident surgical competency. While independent resident operating is essential for skill development, its safety in complex trauma cases remains debated. This study evaluated whether senior residents can safely perform femoral IMN without direct supervision compared with fellowship-trained trauma surgeons.

**Materials and methods:**

A retrospective cohort analysis was performed of adult patients undergoing IMN for isolated femoral shaft fractures between 2015 and 2023 at a level I trauma center. Cases were grouped by primary surgeon: senior residents (postgraduate year ≥ 4) versus attending trauma surgeons. Demographics, fracture characteristics, operative parameters, complications, and revision rates were compared. To minimize selection bias, 1:1 propensity score matching (PSM) was performed based on preoperative covariates including age, comorbidity, fracture type, injury mechanism, and severity.

**Results:**

A total of 207 patients met inclusion criteria; 150 (75 per group) were matched. Operative duration was longer in resident-led cases (median 120 vs. 91 min, *p* = 0.003). Postoperative hospital stay (5.0 vs. 6.0 days, *p* = 0.674) and weight-bearing protocols (*p* = 0.218) were similar. Overall complication rates did not differ significantly (12.0% residents vs. 20.0% attendings, *p* = 0.265), nor did revision rates (20.0% vs. 18.7%, *p* = 0.841).

**Conclusion:**

When appropriately trained and selected, orthopedic residents can independently perform femoral shaft IMN with clinical outcomes comparable to fellowship-trained trauma surgeons. Structured autonomy programs emphasizing competency-based progression allow residents to achieve operative independence in complex trauma procedures without compromising patient safety.

**Level of evidence:**

Level III - a retrospective cohort design.

## Introduction

Orthopedic surgical training follows an apprenticeship model in which residents gradually gain operative experience and autonomy. Balancing comprehensive training with patient safety remains a persistent challenge [1–6]. Studies examining resident participation have yielded mixed results, some reporting longer operative times and higher complication rates [7, 8], while others found no differences in morbidity or mortality [9–11].

Most prior work has assessed surgeries performed with attending supervision, whereas few studies have directly compared outcomes of procedures performed entirely by residents versus attendings. Recent investigations in patellar [4] and ankle [5] fracture surgeries demonstrated comparable short-term results, despite longer operative times for residents, reflecting the educational nature of residency.

However, data remain limited regarding resident autonomy in managing high-energy diaphyseal fractures. Intramedullary nailing (IMN) of femoral shaft fractures is a cornerstone of trauma surgery and a key milestone in operative training, requiring precise reduction, image-guided instrumentation, and timely execution under demanding conditions.

This study compares outcomes of femoral shaft IMN performed independently by residents versus fellowship-trained trauma surgeons to determine whether unsupervised resident performance compromises safety or clinical results, thereby informing the ongoing discussion on surgical education and autonomy.

## Methods

### Patient selection and data collection

This retrospective cohort study was conducted at a level I trauma center serving over two million individuals, with institutional review board approval (5165-18-SMC). Adult patients (≥ 18 years) who underwent surgery for isolated femoral shaft fractures between January 1, 2015, and December 31, 2023, were identified using ICD-10 and CPT codes. Exclusion criteria included pathological, periprosthetic, or polytrauma cases, fractures managed with plating or external fixation, and follow-up < 6 months.

Collected variables included demographics (age, sex, BMI, Charlson Comorbidity Index, smoking status), injury mechanism (low- vs. high-energy), AO/OTA fracture classification (32-A, 32-B, 32-C), and associated factors (bilateral fractures, ipsilateral TKR, open fractures). Injury Severity Score (ISS) was categorized as low (0–15), intermediate (15–25), or high (> 25). Trauma bay labs (lactate, hemoglobin) and perioperative transfusion requirements were recorded. Hospital data included postoperative length of stay and discharge weight-bearing status (NWB, PWB, FWB). Operative details encompassed duration, approach (antegrade or retrograde), acute versus conversion IMN, need for open reduction, cerclage use, and intraoperative “call for help.” Resident postgraduate year (PGY) level was noted for resident-led cases. Data were extracted from electronic records by blinded reviewers.

### Group allocation

Patients were grouped by primary surgeon based on initial intention. The Resident group comprised surgeries initiated independently by a PGY-4 or higher without an attending present. If an attending was subsequently called into the operating room to assist during a resident-led case, the patient was retained in the Resident group for analysis to avoid selection bias.

### Surgical technique

All cases utilized antegrade or retrograde IMN based on fracture location, anatomy, and surgeon discretion. For the antegrade approach, patients were positioned supine on a traction table with a trochanteric entry point; for the retrograde approach, supine with 30–45° knee flexion and intercondylar entry under fluoroscopy. Closed or mini-open reduction was preferred; open reduction was performed if necessary. Sequential reaming preceded insertion of a statically locked intramedullary nail with fluoroscopic verification of alignment. Implant selection was based on availability and did not differ between groups.

### Outcome measures

Primary outcomes were surgical complications. Major complications included nonunion [12], deep infection [13], coronal or sagittal malalignment > 5° (assessed via plain radiographs), malrotation > 15° confirmed by bilateral CT obtained upon clinical suspicion [14], and hardware failure. Minor complications included limb length discrepancy > 2 cm and flexion contracture (extension deficit > 10° or flexion < 90°) [15]. Persistent postoperative pain without mechanical cause was recorded separately as a patient-reported symptom but was excluded from complication rates to maintain consistency with published definitions. Reoperation was analyzed separately and defined as any unplanned return to the operating room related to the index fracture.

Medical complications included superficial infection, VTE, MI, CVA, arrhythmia, pneumonia, CHF, delirium, UTI, ileus, and pressure ulcers. Secondary metrics included operative duration, hospital stay, and time to full weight bearing. Evaluators were blinded to surgeon identity and training level.

### Resident qualification and supervision

The six-year residency program allows independent operating from PGY-4 onward, under a structured autonomy protocol. During this stage, a senior resident may assist, and an attending remains available on call. Each case and plan were reviewed preoperatively with an attending, and patients were informed that a resident might perform the operation. Eligibility for autonomy required successful completion of national-level exams (PGY 1–3), consistent intraoperative evaluations, and a dedicated surgical principles course. Postoperative performance was reviewed in departmental feedback sessions to ensure ongoing quality and safety.

### Statistical analysis

For the initial descriptive analysis of the unmatched cohort, missing values in continuous variables (BMI, lactate, hemoglobin) were imputed using the column median, while missing categorical values (smoking status) were imputed using the mode.

To address baseline confounding and selection bias, 1:1 propensity score matching (PSM) was performed. Propensity scores were generated using a multivariable logistic regression model estimating the probability of being treated by a resident, based on preoperative covariates: age, sex, CCI, mechanism of injury, open fracture, AO/OTA fracture type, bilateral fractures, and ISS category. Matching was conducted using a nearest-neighbor algorithm with a caliper of 0.01. Covariate balance was assessed using standardized mean differences (SMDs), with SMD < 0.1 indicating adequate balance across groups.

Post-matching comparisons were performed using independent-samples t-tests or Mann–Whitney U tests for continuous variables and Pearson’s chi-square or Fisher’s exact tests for categorical variables. Statistical significance was defined as *p* < 0.05. Analyses were conducted using R version 4.2.3 interfaced through Python.

During manuscript preparation, the authors used the ChatGPT language model [GPT-4] to improve readability and subsequently verified and edited all content for accuracy.

## Results

**Fig. 1:**
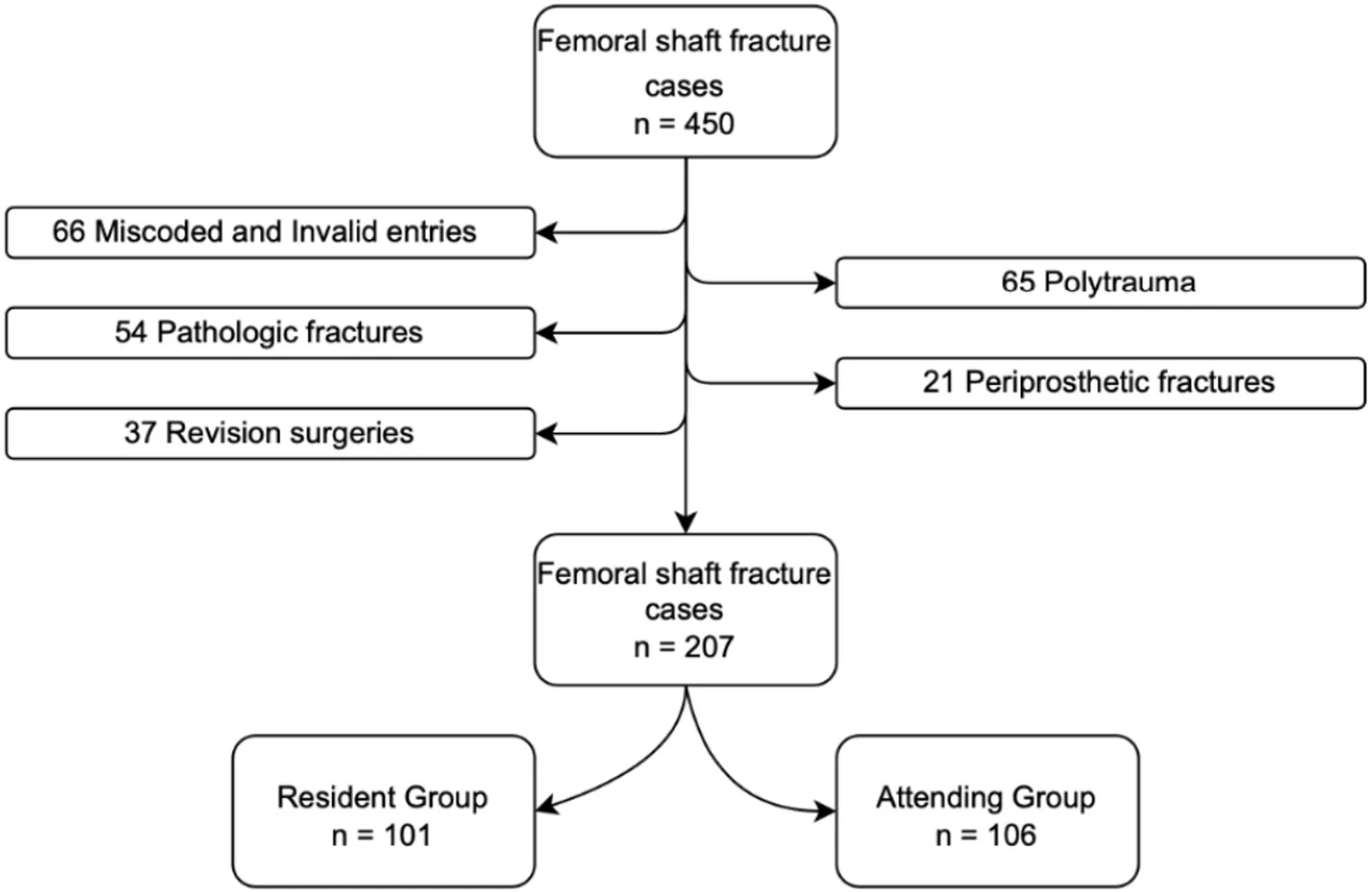
CONSORT Flow Diagram of Patient Inclusion and Exclusion

A total of 450 femoral shaft IMN cases were initially identified. After applying exclusion criteria (polytrauma, pathological, periprosthetic, or revision cases), 207 patients met inclusion criteria: 101 in the resident group and 106 in the attending group [Figure 1]. The minimum follow-up was 6 months (mean = 12.5 months).

### Pre-matching characteristics

Before matching, there were no significant differences in age, sex, BMI, comorbidity burden (CCI), or smoking status [Table 1]. High-energy injuries occurred in 50/101 (49.5%) resident-led cases and 63/106 (59.4%) attending = led cases (*p* = 0.195). Open fractures were seen in 6/101 (5.9%) resident-led cases and 11/106 (10.4%) attending-led cases cases (*p* = 0.353). Complex AO 32-C fractures were more common among attendings (28/106, 26.4%) than residents (11/101, 10.9%) (*p* = 0.015). Severe injury (ISS > 25) occurred in 10/106 (9.4%) attendings versus 2/101 (2.0%) residents (*p* = 0.027).

**Table 1:** Cohort Characteristics

Characteristics		Attendings	Residents		*p*-value
		*n* = 106	*n* = 101		
Patient Characteristics					
Age	[median (IQR)]	46.5 (24.0–76.0)	56.0 (29.0–78.0)		0.207
Gender (F)	[n (%)]	48 (45.3%)	42 (41.6%)		0.692
BMI	[median (IQR)]	24.9 (24.9–24.9)	24.9 (24.9–24.9)		0.982
CCI	[median (IQR)]	0.0 (0.0–4.0)	1.0 (0.0–4.0)		0.418
Smoker	[n (%)]	21 (19.8%)	23 (22.8%)		0.726
Injury Characteristics					
Mechanism of Injury	[n (%)]				0.195
Low Energy		43 (40.6%)	51 (50.5%)		
High Energy		63 (59.4%)	50 (49.5%)		
Open Fracture	[n (%)]	11 (10.4%)	6 (5.9%)		0.353
AO Classification	[n (%)]				0.015*
32-A		54 (50.9%)	65 (64.4%)		
32-B		23 (21.7%)	25 (24.8%)		
32-C		28 (26.4%)	11 (10.9%)		
Bilateral Fracture	[n (%)]	7 (6.6%)	3 (3.0%)		0.332
ISS	[n (%)]				0.027*
Low (0–15)		76 (71.7%)	85 (84.2%)		
Intermediate (15–25)		20 (18.9%)	14 (13.9%)		
High (> 25)		10 (9.4%)	2 (2.0%)		
Lactate	[median (IQR)]	22.0 (20.0-22.4)	22.0 (22.0–25.0)		0.365
Hemoglobin	[median (IQR)]	13.2 (11.8–13.8)	13.2 (12.5–14.1)		0.300
Transfusions	[n (%)]	49 (46.2%)	35 (34.7%)		0.120

**Fig. 2:**
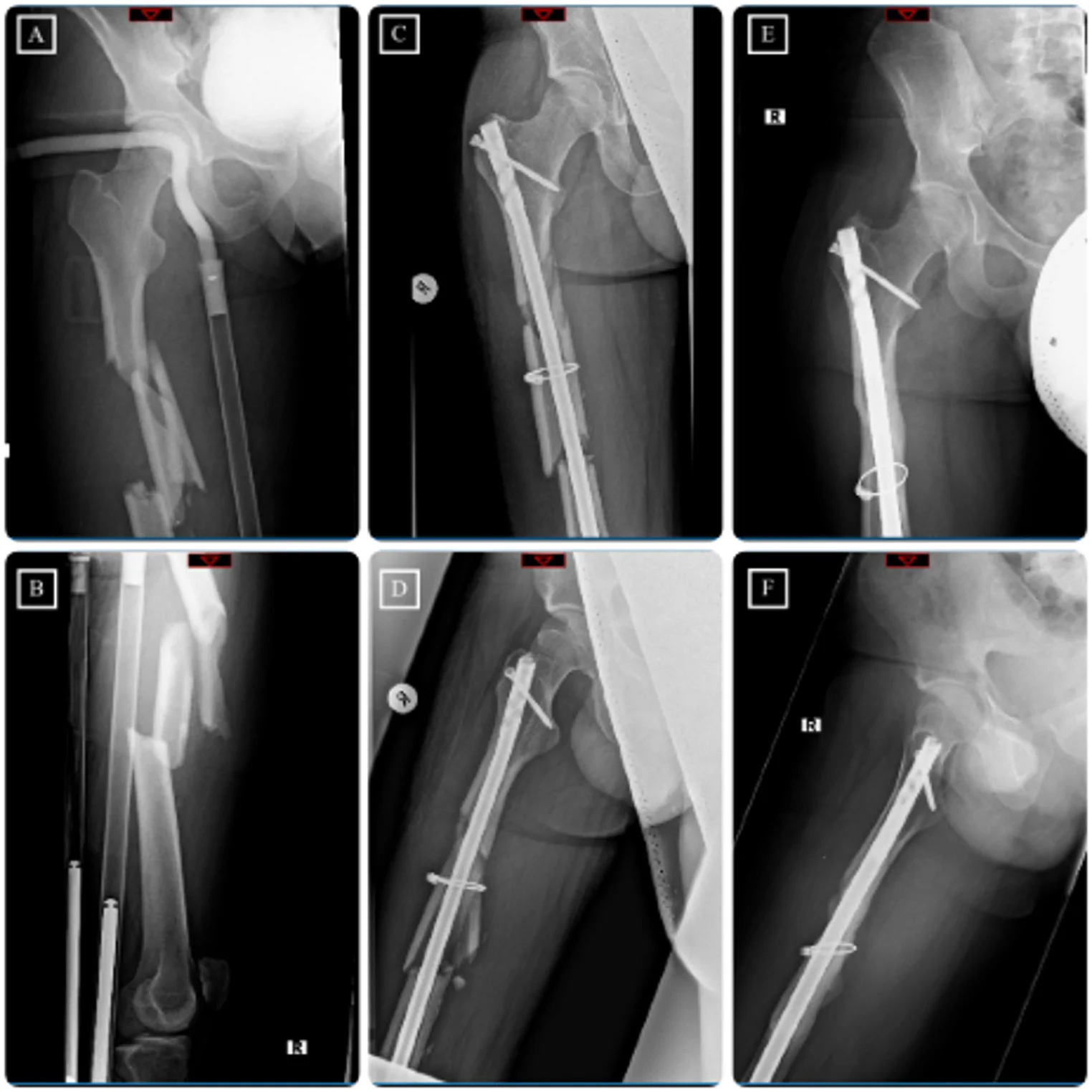
Radiographic Sequence of Midshaft Femur Fracture Treated Primarily by Residents, Complicated by Nonunion and Revised Successfully. **A–B**: Initial injury radiographs showing a closed midshaft femoral fracture.**C–D**: Postoperative images following open reduction and internal fixation with intramedullary nailing and cerclage wiring, performed autonomously by orthopedic residents.**E–F**: Follow-up radiographs one year later, after revision surgery for nonunion, showing radiographic signs of healing and fracture union

Propensity score matching yielded 150 matched patients (75 per group). Covariate balance was achieved across all variables, with standardized mean differences (SMD) < 0.1 except for gender (0.107), bilateral fractures (0.166), and intermediate ISS (0.129), all considered acceptable [Figure 2].

### Operative characteristics (Matched Cohort)

After matching, the groups were comparable in baseline variables. Both groups had an equal rate of acute IMN procedures (69/75, 92.0%; p = 1.000). The antegrade approach was used more frequently by residents (47/75, 62.7%) compared with attendings (34/75, 45.3%) (p = 0.049). Open reductions occurred in 5/75 (6.7%) resident and 12/75 (16.0%) attending cases (p = 0.122). Cerclage wiring was rare, 3/75 (4.0%) resident cases versus none in the attending group (p = 0.239). An intraoperative ‘call for help’ where an attending joined the case was documented in 3 of the resident-led cases (2.9%).

### Outcomes

Post-matching outcomes are presented in Table 2. Median operative time was longer for residents, 120 min (IQR 91.2–150.0), than for attendings, 91 min (IQR 60.0–133.8) (*p* = 0.003). Median postoperative stay was similar between groups (5.0 days [IQR 3–9.5] vs. 6.0 days [IQR 3–9.0]; *p* = 0.674). Weight-bearing protocols did not differ significantly between groups after matching (*p* = 0.218). Among resident-led cases, 30 patients (40.0%) were allowed full weight bearing, 13 (17.3%) partial weight bearing, and 31 (41.3%) remained non-weight bearing. In comparison, attending surgeons permitted full weight bearing in 22 patients (29.3%), partial in 11 (14.7%), and non-weight bearing in 42 (56.0%).

**Table 2:** Post matching outcomes

Outcome		Attendings	Residents	
		*n* = 75	*n* = 75	*p*-value
Surgery Duration	median (IQR)	91.0 (60.0-133.8)	120.0 (91.2–150.0)	0.003*
POD Discharge	median (IQR)	6.0 (3.0–9.0)	5.0 (3.0-9.5)	0.674
Surgical Complications	[n (%)]			
Overall		15 (20.0%)	9 (12.0%)	0.265
Major		11 (14.7%)	6 (8.0%)	0.303
Non-Union		2 (2.7%)	2 (2.7%)	
Infection		7 (9.3%)	2 (2.7%)	
Mal-Rotation		2 (2.7%)	2 (2.7%)	
HW Failure		1 (1.3%)	0 (0.0%)	
Minor		3 (4.0%)	3 (4.0%)	1.000
LLD		1 (1.3%)	3 (4.0%)	
Flexion Contracture		2 (2.7%)	0 (0.0%)	
Revisions	[n (%)]	14 (18.7%)	15 (20.0%)	0.172
Medical Complications	[n (%)]	5 (6.6%)	6 (8.0%)	1.000

Overall surgical complications occurred in 9/75 (12.0%) resident-led and 15/75 (20.0%) attending-led cases (*p* = 0.265). Major complications were observed in 6/75 (8.0%) residents and 11/75 (14.7%) attendings (*p* = 0.303), including nonunion (2 vs. 2), deep infection (2 vs. 7), malrotation > 15° (2 vs. 2), and hardware failure (0 vs. 1).

Minor complications occurred in 3/75 (4.0%) in each group (*p* = 1.000), including limb length discrepancy > 2 cm (3 vs. 1) and flexion contracture (0 vs. 2). There were no instances of coronal or sagittal plane malalignment > 5° in either group.

Postoperative residual pain without identifiable mechanical cause was reported by 21/75 (28.0%) residents and 13/75 (17.3%) attendings (*p* = 0.172). Because of its subjective nature and inconsistent definition in prior literature, it was excluded from complication counts but analyzed separately.

Revisions were required in 15/75 (20.0%) resident-led and 14/75 (18.7%) attending-led cases (*p* = 0.172). The indications for revision in the resident group included elective hardware removal for residual pain (*n* = 8), nonunion (*n* = 2), malrotation (*n* = 2), LLD (*n* = 1), and infection (*n* = 2). In the attending group, indications for revision included infection (*n* = 3), elective hardware removal for residual pain (*n* = 8), nonunion (*n* = 1), malrotation (*n* = 1), and hardware failure (*n* = 1).

**Fig. 3:**
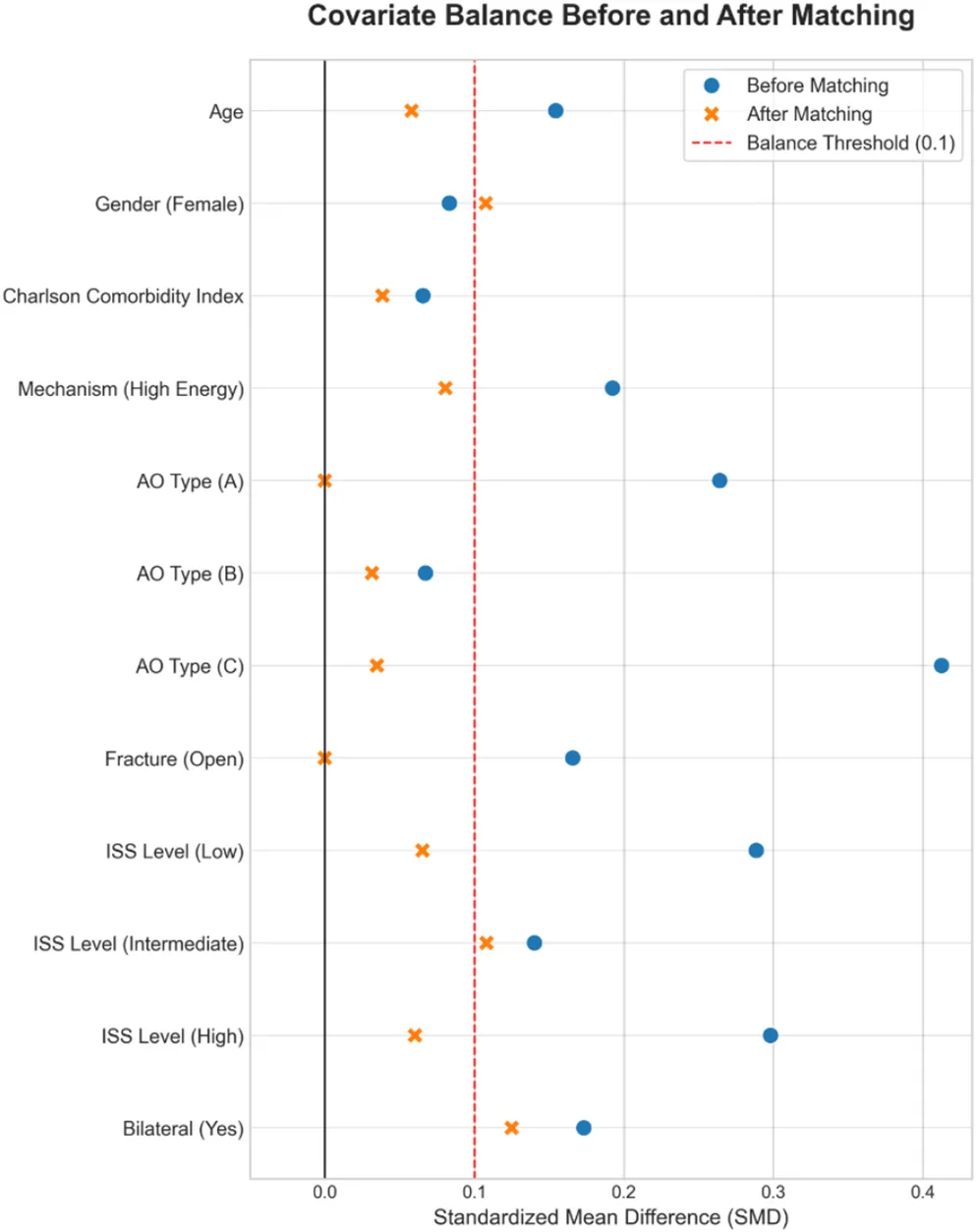
Covariate Balance Before and After Matching

Medical (non-surgical) complications were infrequent with 6/75 (8.0%) in the resident group and 5/75 (6.6%) in the attending group (*p* = 1.000). Figure 3 demonstrates an illustrative case of resident-performed femoral IMN complicated by nonunion and subsequently revised to union.

## Discussion

This study assessed the safety and efficacy of independent resident performance of femoral shaft intramedullary nailing (IMN) compared with fellowship-trained trauma surgeons. As a cornerstone procedure in orthopedic trauma, IMN provides an excellent model for evaluating operative autonomy and readiness for independent practice. The procedure integrates essential competencies in reduction, fluoroscopic guidance, and precise implant placement, making it a critical milestone in surgical training [1].

Before matching, the attending group treated more complex injuries, with a higher proportion of AO type 32-C fractures and greater Injury Severity Scores. This reflects typical triage patterns, where complex or polytrauma cases are preferentially assigned to fellowship-trained trauma surgeons. Propensity score matching was therefore employed to balance patient demographics, injury characteristics, and fracture complexity. This method improved comparability between groups and strengthened the validity of outcome comparisons by minimizing selection bias inherent to retrospective designs [3, 6].

Following matching, operative time remained significantly longer in the resident group. This finding is consistent with previous studies showing that resident participation is associated with increased surgical duration across various orthopedic procedures [1, 2, 4–7, 10]. The prolongation likely reflects both the learning curve and the deliberate pacing of resident-performed surgeries within a teaching environment. Importantly, longer operative times did not translate into higher complication or revision rates, underscoring that patient safety was not compromised.

Postoperative management, including weight-bearing protocols, did not differ significantly between groups after matching. Although modern trends often favor early weight-bearing, a relatively high proportion of patients in this cohort were restricted to non-weight bearing. This conservative approach was primarily driven by the incidence of complex, comminuted fracture patterns (AO type 32-C) requiring protected weight bearing, as well as institutional and surgeon-specific preferences that favored conservative rehabilitation during the earlier years of the study period. This suggests consistent postoperative decision-making between resident and attending surgeons when case characteristics are comparable.

Complication and revision rates were similar between groups, indicating that appropriately trained residents can perform IMN safely and effectively in selected cases. These findings align with previous orthopedic studies showing that, with proper supervision frameworks and structured training progression, resident-led procedures can achieve outcomes equivalent to those of attending surgeons [1, 2, 7]. The results support the educational principle that autonomy should be granted based on demonstrated competency rather than seniority alone.

When evaluating the safety of resident autonomy, analyzing the underlying indications for revision is just as critical as the overall reoperation rate. As detailed in our results, the etiologies driving reoperation were comparable between groups, and importantly, independent resident performance did not result in a disproportionate increase in revisions due to acute technical errors. The incidence of structural or biological failures requiring revision—such as hardware failure, clinically significant malrotation, and nonunion—remained similar between groups. A substantial proportion of the reoperations, particularly within the resident cohort, were elective procedures for hardware removal indicated by late residual pain following successful fracture union. The profile of these revisions reinforces that appropriately trained senior residents can achieve stable, viable fixations without introducing new technical failure modes.

Hospital length of stay was comparable between groups, further reinforcing that resident-led procedures did not negatively influence early recovery or discharge planning. The comparable postoperative course supports the notion that resident autonomy can be implemented safely in a structured and carefully monitored training environment.

Several limitations should be acknowledged. The retrospective design is inherently susceptible to selection bias, although the use of propensity score matching mitigated this concern. Case allocation was not randomized and likely reflected attending discretion based on fracture complexity and patient factors. Furthermore, the retrospective reliance on operative reports likely underestimates the true frequency of intraoperative ‘calls for help,’ as only cases in which the attending physically joined the operation are consistently documented, while informal or remote consultations (e.g., telephone-based review of imaging) are not routinely recorded. The single-center nature of the study, with a well-established residency program, may limit generalizability to other training environments. While the mean follow-up was 12.5 months, the minimum follow-up threshold of 6 months remains a limitation. This duration is sufficient for early postoperative outcomes but will fail to capture all late complications, most notably nonunion or late implant failure. Finally, postoperative CT for rotational assessment was performed selectively and may underestimate the true incidence of malrotation.

Overall, this study supports the feasibility and safety of independent resident performance of femoral IMN when appropriate training, case selection, and supervision frameworks are in place. Structured autonomy models that emphasize competency-based progression appear to balance surgical education with optimal patient care.

## Conclusions

This study demonstrates that orthopedic residents, when adequately trained and carefully selected for independent cases, can perform femoral shaft intramedullary nailing with outcomes comparable to those of fellowship-trained trauma surgeons. Although operative times were longer in the resident group, complication, revision, and hospital stay rates remained equivalent after propensity score matching for fracture complexity, injury severity, and other confounding factors.

These findings support the safety and educational value of structured autonomy programs that grant residents progressive responsibility based on demonstrated competency. When implemented within a supervised and competency-based framework, resident-led procedures can provide meaningful operative experience in complex trauma care without compromising patient safety or quality of outcomes.

## Data Availability

No datasets were generated or analysed during the current study.
